# Flavonoid Glycosides Isolated from Unique Legume Plant Extracts as Novel Inhibitors of Xanthine Oxidase

**DOI:** 10.1371/journal.pone.0032214

**Published:** 2012-03-02

**Authors:** Chrysoula Spanou, Aristidis S. Veskoukis, Thalia Kerasioti, Maria Kontou, Apostolos Angelis, Nektarios Aligiannis, Alexios-Leandros Skaltsounis, Dimitrios Kouretas

**Affiliations:** 1 Department of Biochemistry and Biotechnology, University of Thessaly, Larissa, Greece; 2 Division of Pharmacognosy and Natural Product Chemistry, School of Pharmacy, University of Athens, Athens, Greece; University of Bradford, United Kingdom

## Abstract

Legumes and the polyphenolic compounds present in them have gained a lot of interest due to their beneficial health implications. Dietary polyphenolic compounds, especially flavonoids, exert antioxidant properties and are potent inhibitors of xanthine oxidase (XO) activity. XO is the main contributor of free radicals during exercise but it is also involved in pathogenesis of several diseases such as vascular disorders, cancer and gout. In order to discover new natural, dietary XO inhibitors, some polyphenolic fractions and pure compounds isolated from two legume plant extracts were tested for their effects on XO activity. The fractions isolated from both *Vicia faba* and *Lotus edulis* plant extracts were potent inhibitors of XO with IC_50_ values range from 40–135 µg/mL and 55–260 µg/mL, respectively. All the pure polyphenolic compounds inhibited XO and their K_i_ values ranged from 13–767 µM. Ten of the compounds followed the non competitive inhibitory model whereas one of them was a competitive inhibitor. These findings indicate that flavonoid isolates from legume plant extracts are novel, natural XO inhibitors. Their mode of action is under investigation in order to examine their potential in drug design for diseases related to overwhelming XO action.

## Introduction

Xanthine oxidase (XO) is a flavoprotein, which belongs to molybdenum hydroxylase superfamily and consists of two identical subunits of 145 kDa. Each subunit of the molecule is composed of an N-terminal 20-kDa domain containing two iron-sulfur clusters, a central 40-kDa FAD-binding domain and a C-terminal 85-kDa molybdopterin-binding domain with the four redox centers aligned in an almost linear fashion. Its active form is a homodimer of 290 kDa with each of the monomers acting independently in catalysis [1]. XO is a cytosolic enzyme present in various species, namely bacteria, higher plants, invertebrates and vertebrates [2]. It is also present in several mammalian tissues such as liver, intestine, kidney, lungs, myocardium, brain, plasma and erythrocytes. Among them, XO activity is highest in liver and intestine [3].

XO is the enzyme, which participates in purine degradation, is the main contributor of free radicals during exercise [4], [5]. It uses molecular oxygen as the electron acceptor thereby resulting in production of superoxide radical (O_2_^•−^) and hydrogen peroxide (H_2_O_2_) [4]. However, XO also results in uric acid production which constitutes the most abundant antioxidant molecule in plasma. Thus, the role of XO in redox status is unequivocal since its activity leads to the production of both free radicals and uric acid. Furthermore, XO exhibits a broad specificity toward oxidation of a wide variety of heterocyclic compounds such as purines and pteridines [6], [7] and numerous aliphatic and aromatic aldehydes to the corresponding carboxylic acid [8], [9]. Therefore, it participates in the detoxification of endogenous compounds and xenobiotics.

XO is considered as a major contributor of free radicals in various pathological conditions. More specifically, XO has been implicated in several diseases including ischemia-reperfusion injury, myocardial infarction, hypertension, atherosclerosis, diabetes and cancer [1]. As it has been previously stated XO results not only in free radical production but also in uric acid generation. Gout is a condition where excessive uric acid formation leads to its crystallization and deposition of uric acid crystals in the joints, the connective tissues and the kidneys [10]. Thus, the inhibition of XO activity may have simultaneously antiradical and inhibitory properties with therapeutic interest. The most commonly used and well studied XO inhibitor is allopurinol [11], [12].

Allopurinol [4-hydroxypyrazolo (3,4-d) pyrimidine] is a structural analogue of hypoxanthine [13]. It inhibits the conversion of hypoxanthine to xanthine to uric acid thus decreasing uric acid concentration. It is the only specific competitive, non natural XO inhibitor and is widely used as a drug. Moreover, due to its property to inhibit O_2_^•−^ production, via XO inhibition, allopurinol is considered as a potent antioxidant [5]. However, this is controversial because allopurinol is also considered as a prooxidant molecule because it leads to inhibition of uric acid production as well [14].

Recently, a lot of research has been conducted in order to discover new, natural and specific XO inhibitors [1]. Various plant extracts [15], [16] and polyphenolic compounds, especially flavonoids [17], [18], have been previously examined for their inhibitory properties against XO activity. Legumes constitute an important source of polyphenols including flavonoids (kaempferol, quercetin, anthocyanins and tannins), flavonoid glycosides, isoflavones, phenolic acids and lignans [19], [20].

In a previous study in our research group, several extracts derived from *Leguminosae* family plants cultivated in Greece have been studied for their antioxidant and chemopreventive properties [21]. More specifically, *Leguminosae* family plant extracts and 14 fractions rich in polyphenolic compounds isolated from 2 of them exhibited potent antiradical and chemopreventive properties and protected DNA against free radical-induced damage [21], [22]. In extending these studies, we examined the effects of some of the aforementioned extracts on XO activity. From the results obtained, the extracts exhibited potent inhibitory activity on XO implying that polyphenols present in them are responsible for their biological properties [23]. Our previous results imply that these specific plant extracts are a possible source of new natural XO inhibitors. Thus, in the present study we examined the inhibitory activity of the 14 fractions and pure polyphenolic compounds isolated from them on XO.

## Materials and Methods

There were no specific permits were required for the described field studies. In addition, no specific permissions were required for the collection of the plants, where the extracts were obtained. This is because the studied legume plants are very common in Greece and they are not cultivated in privately-owned or protected areas. Finally, the field studies did not involve endangered or protected species.

### Chemicals and reagents

Xanthine oxidase from bovine milk and allopurinol were purchased from Sigma-Aldrich (St Louis MO, USA). All the other chemicals, KH_2_PO_4_, Na_2_HPO_4_2H_2_O and EDTA, were purchased from Sigma-Aldrich (St Louis MO, USA) and were of the highest quality (≥99.0%) commercially available.

### Plant material

The edible plants *V. faba* and *L. edulis* (*Leguminosae*) were cultivated in a parcel near to village ‘Zaros’ of the island Crete (Greece). After harvesting (2005), the aerial parts of the plants (leaves and branches) were transferred to the laboratory and left to dry under shadow at room temperature. The air-dried aerial plant parts were pulverized by a mill machine (Allenwest, Brighton, England).

### Extraction, fractionation and identification of polyphenolic isolates

The extraction, fractionation and identification of the polyphenolic fractions has been previously described [21]. Thin-layer chromatography (TLC) was carried out on glass pre-coated silica gel 60 F_254_ plates (Merck). Fast centrifugal partition chromatography (FCPC) was performed using a CPC KROMATON with a 1000 mL column, adjustable rotation of 200–2000 rpm and a Lab Alliance pump with a pressure safety limit of 50 bars. A manual sample injection valve was used to introduce the samples into the column. A Τhermo Finnigan HPLC instrument was employed making use of a SpectraSystem P4000 pump, SpectraSystem 1000 degasser, SpectraSystem AS3000 automated injector and a UV SpectralSystem UV2000 detector. NMR spectra were obtained with a Bruker AC200 (200 MHz) and a Bruker DRX 400 (400 MHz) spectrometer. Chemical shifts were given as *δ* values with TMS as the internal standard. The 2-D experiments (COSY, COSY LR, HMQC, HMBC, TOCSY and NOESY) were performed using standard Brucker micro programs. ESMS (*Electron Spray Mass Spectrometry*) were recorded with a Nermag R 10 10 C apparatus.

In details, the powder of the aerial parts of *V. faba* (0.890 Kg) and *L. edulis* (0.490 Kg) were extracted with CH_2_Cl_2_ (dichloromethane), MeOH (methanol) and H_2_O (water), successively. Each solvent extraction (3×5 L) was repeated three times, for 48 h per extraction. The MeOH extract of *V. faba* (48.1 g) was processed with resin XAD-4 in purpose to separate the total of phenolic compounds (12.4 g). A part of this fraction (12 g) was subjected to CPC separation using a mixture of EtOAc∶ *n*-BuOH∶ H_2_O (2∶1∶3) as biphasic system. The separation was run at a revolution speed of 1000 rpm. Initially, the lower phase was used as the mobile phase (water based), while the upper phase was used as the stationary phase in a head to tail or descending mode (flow rate 15 mL/min). The effluent of the column was manually collected in 30 mL aliquots and this procedure resulted in eleven fractions (Vf A–L). Then, the organic layer in a tail to head or ascending mode and five fractions (Vf M–Q) were collected.

Similarly, a part of the *L. edulis* MeOH extract (12 g) was subjected to CPC separation using a mixture of heptane∶ EtOAc∶ MeOH∶ H_2_O (1∶4∶1∶4) as biphasic system. The separation was run at a revolution speed of 1100 rpm. Initially, the upper layer of the biphasic system was used as the mobile phase, while the lower layer (water based) was used as the stationary phase in a tail to head or ascending mode (flow rate 15 mL/min). The effluent of the column was manually collected in 30 mL aliquots and this procedure resulted in nine fractions (Le A–I). Then, the water layer was used as mobile phase in a head to tail or descending mode and six fractions (Le K–P) were collected.

**Figure 1:**
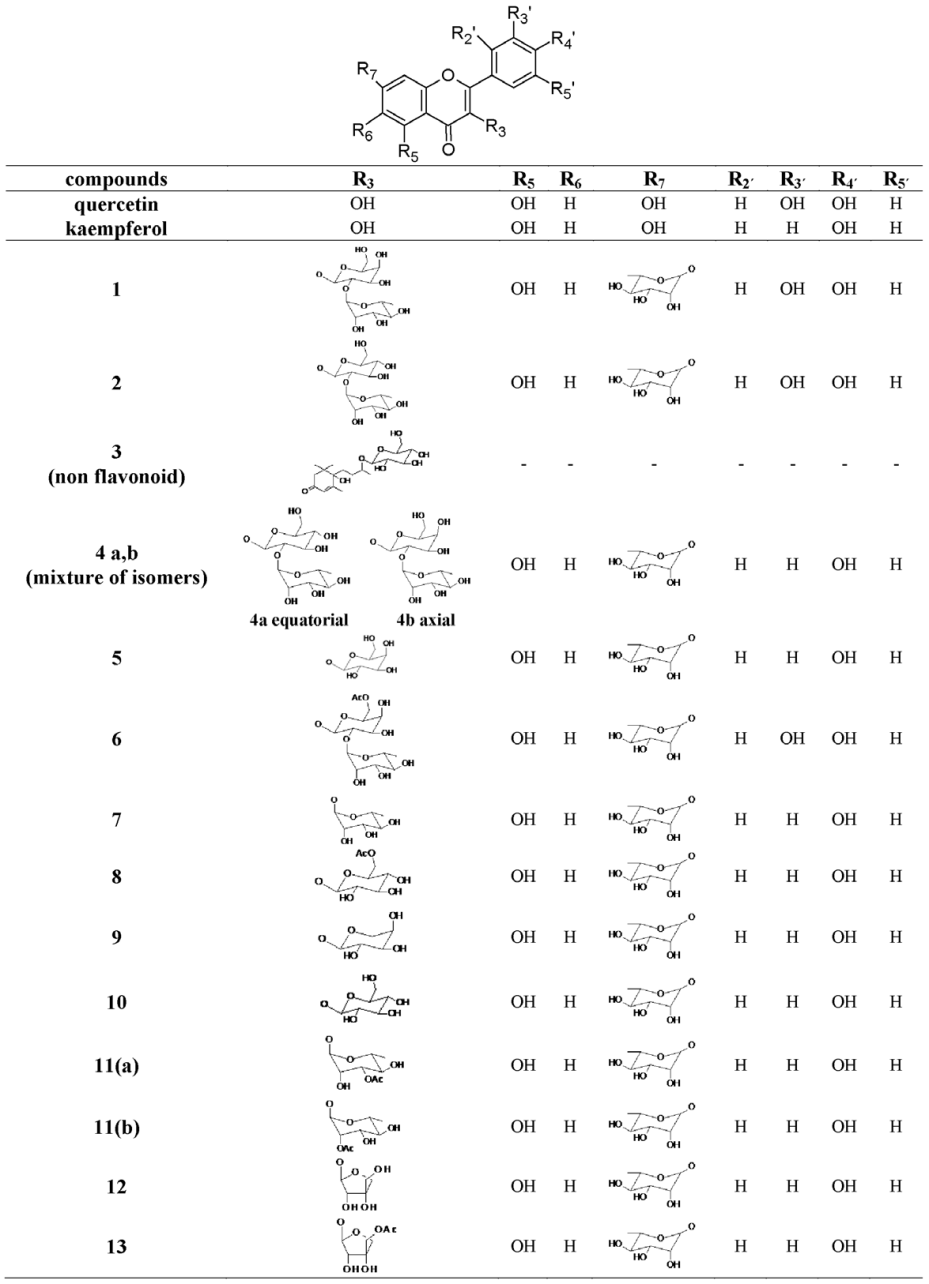
Chemical structures of the polyphenolic compounds isolated from the legume plant extracts.

All fractions isolated from both plants, were examined by thin layer chromatography (TLC) and analytical high performance liquid chromatography (HPLC) using a 250×4.0 mm i.d Lichrosorb RP18 column (BIRSHOFF Chromatography, Loenberg, Germany). The mobile phase was a linear gradient of 2% aqueous acetic acid (solvent A) and acetonitrile (solvent B). Conditions: initial A–B (95∶5); in 70 min A–B (75∶25); back to initial conditions; flow rate 1 mL/min. Further purification of compounds of interest was accomplished using preparative HPLC. All compounds were structurally elucidated using 1D and 2D NMR spectroscopy as well as UV and MS techniques (Fig. 1). All the known compounds (**1–11**) were confirmed by comparison to literature data [24]–[39]. Compounds **12** and **13** were new and their characterization was described in a previous study of ours [21].

### Fractions and pure polyphenolic compounds

Ten fractions were isolated from the aerial parts extract of *V. faba* and four from the extract of *L. edulis* (Table 1). Fourteen polyphenolic compounds were identified in the fractions and apart from compound **3** they are kaempferol and quercetin glycosides. The structure of the compounds that constitute each fraction is depicted in Fig. 1 and classified according to the basic flavonoid structure. More specifically the compounds identified are: Compound **1**: Quercetin 3-*O*-[*α*-L-rhamnopyranosyl(1→2)-*β*-D-galactopyranosyl]-7-*O*-*α*-L-rhamnopyranoside; Compound **2**: Quercetin 3-*O*[*α*-L-rhamnopyranosyl(1→2)-*β*-D-glucopyranosyl]-7-*O*-*α*-L-rhamnopyranoside; Compound **3**: (6S,9R)-Roseoside; Compound **4 (a,b isomers)**: Kaempferol 3-*O*-[*α*-L-rhamnopyranosyl(1→2)-*β*-D-glucopyranosyl]-7-*O*-*α*-L-rhamnopyranoside and Kaempferol 3-*O*-[*α*-L-rhamnopyranosyl(1→2)-*β*-D-galactopyranosyl]-7-*O*-*α*-L-rhamnopyranoside; Compound **5**: **5**: Kaempferol 3-*O-β*-D-galactopyranosyl-7-*O-α*-L-rhamnopyranoside; Compound **6**: Quercetin 3-*O*-[*α*-L-rhamnopyranosyl(1→2)-6-*O*-acetyl-*β*-D-galactopyranosyl]-7-*O*-*α*-L-rhamnopyranoside; Compound **7**: Kaempferol 3-*O-α*-L-rhamnopyranosyl-7-*O-α*-L-rhamnopyranoside; Compound **8**: Kaempferol 3-*O*-(6-*O*-*β*-D-acetylglucopyranosyl)-7-*O*-*α*-L-rhamnopyranoside; Compound **9**: Kaempferol 3-*O*-*α*-L-arabinopyranosyl-7-*O*-*α*-L-rhamnopyranoside; Compound **10**: Kaempferol 3-*O-β*-D-glucopyranosyl-7-*O-α*-L-rhamnopyranoside; Compound **11a**: Kaempferol 3-*O*-(3-*O*-acetyl-*α*-L-rhamnopyranosyl)-7-*O*-*α*-L-rhamnopyranoside; Compound **11b**: Kaempferol 3-*O*-(2-*O*-acetyl-*α*-L-rhamnopyranosyl)-7-*O*-*α*-L-rhamnopyranoside; Compound **12**: Kaempferol 3-*O-β*-D-apiofuranosyl-7-*O-α*-L-rhamnopyranoside; Compound **13**: Kaempferol 3-*O*-(5-*O*-acetyl-*β*-D-apiofuranosyl)-7-*O-α*-L-rhamnopyranoside.

**Table 1:** IC_50_ values and composition of the polyphenolic fractions isolated from the methanolic extracts of aerial plant parts of *V. faba* and *L. edulis*.

Fractions from*V. faba* extract	IC_50_(µg/mL)	Compounds	Compounds ratio
Vf N	40	7∶8	2∶1
Vf P	55	9∶10	1∶1
Vf E	66	3∶4	3∶1
Vf I	72	5∶6	1∶4
Vf K	72	5∶10∶6	4∶1∶2
Vf C	85	1∶2	2∶1
Vf L	94	mixture of non polar compounds	
Vf Q	95	5∶10	2∶3
Vf F	115	3∶4	2∶1
Vf B	135	1∶2∶3	3∶1∶3
Meth. *V. faba* extract	540		

Fractions from*L. edulis* extract	IC_50_(µg/mL)	Compounds	Compounds ratio
Le E	55	11 a,b (isomers)	1∶1
Le O	90	12	1
Le B	97	13	1
Le N	260	7	1
Meth. *L. edulis* extract	410		

The composition of the fractions with the aforementioned compounds is presented in Table 1. Only fraction Vf L is consisted of unidentified non polar compounds. On the contrary with *V. faba* fractions, which were consisted of a combination of phenolic compounds, *L. edulis* fractions were mainly consisted of pure flavonoid compounds (Table 1). Only Le E fraction was consisted of a mixture of two isomers of compound **11**, **11a** and **11b** (Table 1).

### Assessment of XO activity

XO activity was determined by measuring the formation of uric acid from xanthine within a set time. Changes in the levels of uric acid produced through the presence of the test samples allow the potency of inhibitors to be assessed. Initially the effect of the polyphenolic fractions and compounds on the inhibition of enzymatic activity was assessed at substrate concentration where maximum velocity was obtained. All initial reaction rates were in the linear scale and were measured over the first 4 min of the reaction. Each assay was performed in triplicates and the optical density was measured using a Hitachi U-1500 Spectrophotometer (San Jose, USA). The reaction mixture (500 µL) contained sodium phosphate buffer (33 mM pH 7.5 with EDTA 0.1 mM), xanthine (4.8 µM) and the extract in various concentrations. The reaction was initiated through the addition of 43 mU unit of XO diluted in H_2_O. The production of uric acid was determined at room temperature at 295 nm for 4 min. Control samples were prepared identically to the test samples without the addition of the specimen.

The IC_50_ values were determined as the concentration of the fractions or compounds required to inhibit XO activity by 50% monitored through the decrease in uric acid production. Allopurinol was used as positive control since it is a known inhibitor of XO [12]. The measured absorbance was corrected for the contribution of the tested fractions and the pure polyphenolic compounds.

### Statistical analysis

Statistical computations were carried out using SPSS 13.0 software. For statistical analysis, one-way ANOVA was applied followed by Dunnett's test for multiple pair-wise comparisons. Differences were considered significant at *p*<0.05. The steady state kinetic parameters (K_m_ and V_max_) were determined (under standard assays conditions) by analyzing formation of uric acid from xanthine under initial rate conditions using the Lineweaver-Burk linearization of the Micaelis-Menten equation. The K_i_ values and the apparent kinetic parameters K_m_ and V_max_ were analyzed with the statistic package Grafit 6 (Erithacus Software, Ltd., Horley, UK).

## Results

### Inhibitory activity of the polyphenolic fractions

All the fractions isolated form both *V. faba* and *L. edulis* plant extracts were potent inhibitors of XO. IC_50_ values range from 40–135 µg/mL and 55–260 µg/mL for *V. faba* and *L. edulis* fractions, respectively (Table 1). All the polyphenolic fractions showed higher XO inhibition than that of the initial plant extracts (Table 1). According to the results of *V. faba* fractions, Vf N exerted the most potent inhibitory activity on XO whereas Vf B was the less potent. Regarding *L. edulis*, Le E and Le N were the most and the less potent fractions, respectively.

### Inhibitory activity of the pure polyphenolic compounds

From the 14 identified compounds only 11 were tested for their inhibitory activity on XO (Table 2). More specifically, the compounds **6** and **8** were not isolated because they were detected in very low quantities in the fractions (Fig. 1). Additionally, the compounds **11a** and **11b** could not be separated from the fraction Le E (Table 1, Fig. 1).

**Table 2:** Inhibition type and K_i_ values of the pure compounds.

Pure compounds	K_i_ (µM)	Inhibition type
Allopurinol	0.55	Competitive
1	115	Non competitive
2	186	Non competitive
3	767	Non competitive
4a	651	Non competitive
4b	50	Non competitive
5	345	Non competitive
7	296	Non competitive
9	210	Non competitive
10	177	Non competitive
12	215	Non competitive
13	13	Competitive

**Figure 2:**
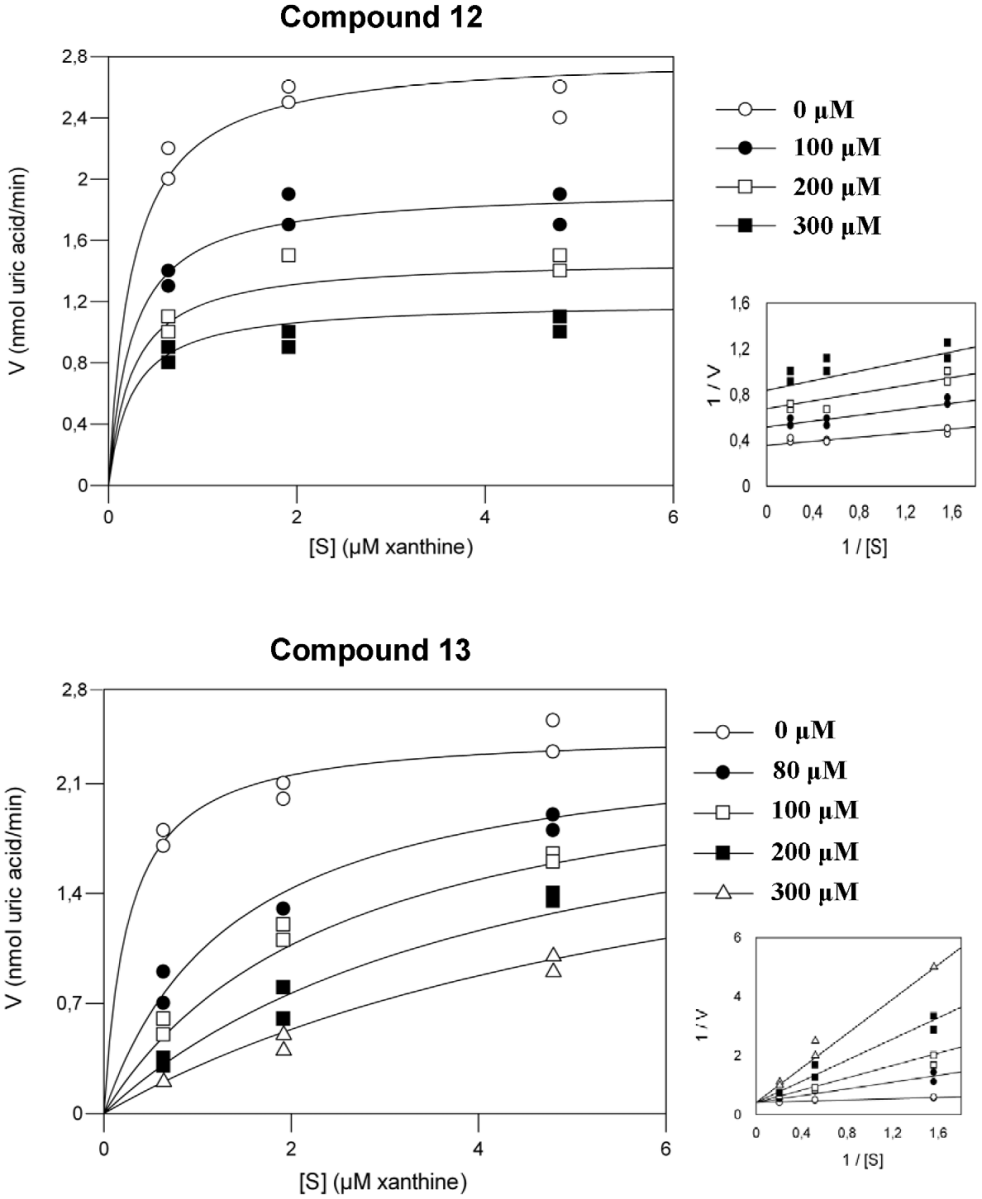
Kinetic analysis plots of the compounds 12 and 13.

All the pure polyphenolic compounds inhibited XO (Table 2). The K_i_ values range from 13–767 µM. According to the kinetic analysis compound **13** was the only competitive and the most potent inhibitor (Fig. 2). The remaining 10 compounds followed the non competitive inhibitory model (Table 2). Compound **4b** was the most potent among them (Table 2) and compound **3** the less potent. Additionally, all the compounds were less potent inhibitors than allopurinol (Table 2).

## Discussion

Legumes have recently gained a lot of interest for the beneficial health implications of their polyphenolic compounds [19], [20]. Dietary polyphenolic compounds, especially flavonoids, except their potent antioxidant activity [39], [40] they are potent inhibitors of XO activity [17], [18]. XO is the main contributor of free radicals during exercise but it is also involved in pathogenesis of several diseases such as vascular disorders, cancer and gout [1], [5], [10]. In order to discover new natural, dietary XO inhibitors, some polyphenolic fractions and pure compounds isolated from two legume plant extracts were tested for their effects on XO activity.

The two methanolic extracts, where the fractions and the polyphenolic compounds are originated from, exhibited potent XO inhibitory activity (Table 1). From the results obtained, all the polyphenolic fractions were more potent than the initial total extracts. The different combinations of polyphenolic compounds present in each fraction could partly explain the different inhibitory efficiency of the fractions (Table 1). The most potent activity of the isolates also implies that the polyphenolic compounds may act differently when they are present in an extract. For example, Vf N which was the most potent polyphenolic fraction had IC_50_ value at 40 µg/mL, whereas the initial *V. faba* plant extract had IC_50_ value at 540 µg/mL. Additionally, their enrichment in the procedure of their final preparation may be another reason for their high activity as compared to their initial fractions.

All the pure polyphenolic compounds inhibited XO and the K_i_ values range from 13 to767 µM (Table 2). Their IC_50_ values, range from 42 to 260 µg/mL implying that the pure compounds exert the same pattern of action either they are pure or as a mixture in a fraction (Table 1). A representative example is the fraction Vf P which is consisted of the compounds **9** and **10** in the same ratio (1∶1). The IC_50_ values of the compounds **9** and **10** are 63 and 42 µg/mL respectively whereas the IC_50_ of the Vf P fraction is 55 µg/mL, demonstrating that there is not a synergistic effect of the two compounds.

It is noteworthy that all potent compounds were flavonol glycosides and more specifically kaempferol and quercetin glycosides. Various flavonoids are known for their inhibitory activity on XO. Regarding the basic chemical structure of flavonols, the hydroxyl groups at C5 and C7 along with the double bond between C2 and C3 were found to be essential for their high inhibitory activity [17], [41]. The compounds tested in the present study have the double bond between C2 and C3 and the unsubstituted hydroxyl group at C5, whereas the hydroxyl groups at C7 and C3 are substituted with the glycosides (Fig. 1). Consequently, the observed inhibitory activity is partly attributed to these characteristics. However, quercetin and kaempferol, the aglycone forms of our compounds, exhibit very low K_i_ values in comparison with our results [18]. Previous studies have demonstrated that the substitution of hydroxyl groups at C7 and C3 of the basic flavonoid structure by a glycoside or a methyl group seems to decrease the XO inhibitory effect [41]. Therefore, the presence of the glycosides is responsible for the lower inhibitory activity of our compounds.

According to the kinetic analysis, compound **13** was the only competitive and the most potent inhibitor (Fig. 2). The compound **3** which is a non flavonoid had the less potent inhibitory activity (Fig. 1). It is noteworthy that compound **13** differs from compound **12** in one acetyl group (-COCH_3_) at its chemical structure. Unexpectedly such a small structural difference may result in the different competition scheme (Table 2) (Fig. 1,2). Similarly, these compounds exhibited different antioxidant and chemopreventive properties (21). Additionally, it is noteworthy despite the fact that compounds **4a** and **4b** constitute isomers of the same compound they exhibited different potency at their inhibitory action (Table 2). It seems that the inhibitory activity of the compounds depends on the position of the hydroxyl group (**4a** equatorial, **4b** axial) at the glycoside substituting the hydroxyl group C3 of the basic flavonoid structure (Fig. 1). Furthermore, compounds **1**, **2**, **9** and **10** were potent non competitive inhibitors. Despite the fact that quercetin glycosides **1** and **2** and kaempferol glycosides **9** and **10** have structure differences in their C3-O-glycoside, they exert similar activities (Table 2, Fig. 1).

In order to elucidate the specific mechanism of inhibition and justify the aforementioned differences, more detailed kinetic analysis and structural work is in progress in order to find the mode of binding of these compounds to the enzyme molecule, since the crystal structure of XO from bovine milk has been previously elucidated [42], [43]. As yet, reports concerning the mode of inhibition of polyphenolic compounds have been contradictory [41].

As mentioned above, allopurinol was used as a positive marker of potent XO inhibitory activity. Indeed, its K_i_ value was about 10 to 1000 fold lower than the K_i_ values of the tested polyphenolic compounds (Table 2). This is due to the fact that allopurinol is a structural isomer of xanthine and a specific competitive inhibitor of XO. The polyphenolic compounds tested are novel, natural XO inhibitors. However the findings of this study serve as preliminary data for further modifications in the structure of the compounds. When the mechanism of XO inhibition is fully explained, there is possibility that some of these molecules may serve as lead compounds with better and more specific inhibitory properties for drug design. These molecules are natural products that possibly lack the side effects of allopurinol [11].

The pleiotropic action of the polyphenolic fractions isolated from the legume extracts have been previously investigated and assessed [21]. More specifically, they possess potent antioxidant and chemopreventive properties against free radical-induced DNA damage [21]. Generally, polyphenols have been established as pleiotropic molecules with various biological properties [44]. Furthermore, XO inhibition is mainly considered as a mechanism of the antioxidant activity of polyphenols as the majority of the studies consider XO as a contributor of free radicals [41], [45]. Thus, these flavonoid isolates and the initial extracts and fractions may be used as antioxidant, nutritional supplements.

Antioxidants and polyphenolic compounds have been widely used as part of human daily diet or as supplements especially before exercise in order to provide protection against exercise-induced oxidative stress or tissue oxidative damage. However, XO inhibition leads not only to decrease of free radical production but also to inhibition of uric acid generation. When oxidative stress is present, uric acid is necessary as potent antioxidant molecule. It is not well established if inhibition of free radical production or uric acid generation is more useful during exercise. Recent evidence of our research group have demonstrated that allopurinol administration, a known antioxidant molecule and XO inhibitor, induced oxidative stress and reduced performance after swimming until exhaustion in rats [14]. In this case, uric acid seems to be essential for ameliorating the harmful effects of exercise.

The findings of this study indicate that flavonoid isolates from fractions derived from legume plant extracts are novel, natural XO inhibitors. Their mode of action is under investigation in order examine their potential in drug design for XO related diseases such as gout. Considering that legumes have beneficial health implications, they may be used as dietary supplements. Nevertheless, regarding that XO inhibition seems to favour oxidative stress their supplementation before exercise should be carefully considered.
